# Abstracts from Foreign Journals

**Published:** 1902-02

**Authors:** M. P. Ravenel, S. J. J. Harger

**Affiliations:** Philadelphia, Pa.; Philadelphia, Pa.


### ABSTRACTS FROM FOREIGN JOURNALS.

### FRENCH.

UNDER THE DIRECTION OF M. P. RAVENEL, M.D., AND S. J. J. HARGER, V.M.D., OF PHILADELPHIA, PA.

MEDIAN NEURECTOMY AND FLEXOR TENOTOMY FOR KNUCKLING. Breton describes three degrees of knuckling: *First degree,* when the first phalanx has not been displaced beyond the vertical; *second degree,* when a perpendicular tangent to the anterior face of the fetlock strikes the hoof; *third degree,* when this perpendicular falls in front of the hoof.

After speaking of the recurrence of knuckling after tenotomy, the sensibility of the cicatrix, and a recurrence of the lameness the author supplements section of the tendon with median neurectomy. The suppression of the sensibility does not seem to interfere with the normal cicatrization of the tendinous wound.

*Operation.* The foot being properly dressed, a shoe with long heel and a long toe-piece with the end curved upward is applied. The subject is cast upon the diseased side and the median nerve resected; he is then turned over and the tendons are cut. The two wounds cicatrize normally and the position of the bones often becomes normal at once. At the end of fifteen days a thin-heeled shoe is applied, and in six weeks a cure is effected. The perforans alone is severed. This treatment is not infallible, but it has given many good results. Perfect results have been obtained in knuckling of the third degree, even when both legs were affected; also, with or without morbid changes accompanying the contraction of the tendons. Asepsis and other surgical directions must be observed.—*Rec. de Med. Vet.*

OPERATIVE MODIFICATION IN NEURECTOMY. After the nerve has been isolated, Bessange recommends its section with a heated metallic blade instead of the bistoury. The blade of the zoo-cautery can be used for this purpose. The author claims that this procedure produces a complete asepsis of the end of the stumps, that the sensibility of the foot from union of the stumps is not so apt to recur, and neuromata are not so frequent. These results are claimed from observation of many cases.—*Rec. de Med. Vet.*

Abscess of the Pleura. The animal, while backing, fell on its side upon a sharp piece of iron, making a lacerated wound 3 to 4 centimetres long on the middle of the side of the chest, between the seventh and eighth ribs. When seen by Pousselet, eighteen days after the accident, the wound was of a fistulous character, discharging a clear, grumous pus on extirpation. A blister had been applied upon both sides of the chest. There was dulness on percussion around the seat of injury, but beyond this area there was normal resonance. Puncture with the trocar revealed the absence of pleuritic exudation.

The fistula was enlarged and curetted, allowing the escape of a quantity of grumous pus, and injected with bichloride and trikresol. A drainage-tube was inserted, but was soon displaced. At the end of a month the wound was again enlarged to about 10 cm., and a pint of infectious pus escaped: the hand was introduced into the cavity to clean out the fibrinous exudation. Inflammatory adhesions had formed between the visceral and parietal pleura, which protected the pleural cavity from infection through the cavity of the abscess and prevented the communication of the former with he atmosphere. Bichloride irrigation was employed for about fifteen minutes after the operation, the cavity was filled with cotton and iodoformed ether, and a bandage applied. The wound eventually entirely closed, leaving only a cicatrix. Constitutional symptoms were at no time marked.—*Rec. de Med. Vet.*

Osteitis of Founder. Since the investigations of Lesbre, which indicate that founder is an osteitis circumscribed to the antero-inferior part of the os pedis, Jacoulet, Joly, Vivien, and Liénaux confirm the real existence of an osteitis with osteophytes. In a horse which was destroyed forty-eight hours after the beginning of the attack, examination showed that the osteitis is extensive; that the entire bone may be involved, even the lateral cartilage and the navicular bone.

In cases of insidious founder primarily chronic or subacute (Jacoulet) osteitis is often the initial stage, although it cannot be said without reserve that such is always the case. This would be precisely opposed to the view, heretofore generally accepted, that the podophyllous tissue is the essential seat of the disease. The latter view, considering the sensibility and the vascularity of the lamina, would offer a better explanation for those acute cases which manifest themselves very suddenly. To harmonize these two views requires further observation.—*Ann. de Med. Vet.*

OBSERVATIONS UPON CRYPTORCHIDISM. Sabot reports the following results of 101 cases: Double abdominal, 11; unilateral abdominal, 79; double inguinal, 3; unilateral inguinal, 8.

Cryptorchidism is most frequent in the left side. The testicle, which is of variable volume, shows, as a rule, no degeneration. Three times serous or sebaceous cysts were found. In five cases the testicle could not be found, and in two of these the autopsy showed the absence of a testicle on account of a malformation of the internal genital organs. Four cases died from the effects of the operation—2 hernia and 2 peritonitis.

It will be seen that abdominal cryptorchidism is more frequent than the inguinal form.—*Rev. Vet.*

SYNOVIAL CYST OF THE KNEE IN THE HORSE. The radio-carpal synovial membrane sometimes forms a synovial tumor above the supracarpal bone, between the external border of the radius and the tendon of the external flexor of the metacarpus. Lesbre treated one of these with the classical iodine method. The articular cavity was emptied with the capillary trocar and the iodine injected. A few hours after the operation the region became hot, painful, and tumefied. In forty-eight hours resolution commenced and was complete in fifteen days.—*Jour. de Med. Vet. et de Zoötschnie.*

TREATMENT OF HÆMOGLOBINURIA (AZOTURIA). As soon as the diagnosis is made, Kas administers 400 to 500 grammes of bromide of potassium in distilled water and bleeds from the jugular vein; 10 centigrammes of sulphate of eserine are given hypodermically. The animal is then rubbed with a stimulating liniment, such as camphorated alcohol, and the hind-quarters are covered with cold compresses, over which several woollen blankets are placed. The bladder is catheterized and the rectum emptied. In grave cases a second dose of the bromide is given the next day. The patient is never placed in slings before the third day. Out of sixteen cases this treatment was successful in fifteen.—*Ann. de Med. Vet.*

FREQUENCY OF TUMORS IN THE HORSE. Observation upon 5000 horses presented at the Berlin clinic from 1895 to 1901 showed, according to Fröhner, 150 cases of tumors, as follows: Sarcoma, 41 (28 per cent.); bothryomycosis, 30 (20 per cent.); fibroma, 20 (13 per cent.); carcinoma, 20 (13 per cent.); keloids,

8 (5 per cent.); keraphyllocele, 8 (5 per cent.); papilloma, 8 (5 per cent.); adenofibroma, 4 (4 per cent.); lipoma, 4 (3 per cent.); osteoma, 2 (1 per cent.); atheroma, 2 (1 per cent.).

These figures do not include tumors of the shoulder, elbow, and testicular cord. In every case a microscopical examination was made.

Among *sarcoma* the melanotic variety predominated (11 upon 28); the anus and rectum were the principal places of election of melanotic sarcoma (4 upon 11). Two were situated upon the sheaths of the penis; five upon the mammary gland, abdomen, elbows, head, and eye.

Fibrosarcoma was almost as frequent as the preceding. Three were found on the superior eyelids and one each on the superior lip, cheek, ethmoid bone, mamma, and sheath of the penis; four round-celled sarcoma were found in the head, breast, thigh, and rectum; two lymphosarcoma in the sublingual and prescapular lymph glands; one osteosarcoma of the superior jaw and another on the zygomatic crest.

A spindle-celled sarcoma of the internal angle of the eye was operated on four times, and during the last year there was no evidence of recurrence.

Of 10 *carcinoma*, 3 occupied the maxillary sinus, 4 the prepuce and the penis (place of election in the horse), and 3 the internal angle of the eye. All belonged to the pavement-celled variety. In one case a cancer of the maxillary sinus perforated the hard palate and penetrated into the mouth. In three cases, involving the sheath and the penis, extirpation of the tumor and the corresponding glands was practised.

Bothryomycosis was seen 16 times. Four were found on the mammary glands, and were successfully removed.

Of the 15 *fibromata*, 5 were found on the posterior, 4 on the abdomen, 3 on the thorax and shoulder; in 3 other cases it was a question of generalized fibromatosis, with localizations upon the internal face of the hind leg, the stomach, and breast. Excision was attended with success.

Of 8 keloids, 5 were found on the coronet and heels, and 3 on the canon. They resulted from calking, and were successfully treated by extirpation and cauterization.

Four cases of adenofibroma on the inferior third of the nasal mucous membrane were observed. They consisted of an agglomeration of small tumors of the size of a walnut, hard or soft, ulcerated and bleeding easily, and covered with a crust; the sublingual lymphatics were intact. Extirpation is made with a sharp instrument and the part curetted. They become more accessible by cutting the external ala of the nostrils and afterward suturing the same. Tracheotomy may be performed.

The osteomas were pediculated and situated on the inferior border of the lower jaw.

*Treatment*. Circular incision around the base, section with a saw, and suturing of the skin and the periosteum.

Two lipomas were seen—one on the forearm and another as large as a child's head in front of the sheath. Treatment, extirpation.—*Ann. de Med. Vet.*

OPOTHERAPY WITH THE JUICE OF THE SYMPATHETIC SYSTEM. Following Brown-Séquard, Boschetti, of the University of Parma, studied opotherapy with the testicular and cerebral juices, and demonstrated that these have a bactericidal action and are of therapeutic value when administered subcutaneously.

Recently, Boschetti has studied the juice of the sympathetic nervous system, which was prepared from the large sympathetic trunks and ganglia taken from horses and cattle. It was administered to dogs suffering from distemper and debility from chronic affections. From his experiments the author believes that this juice, as well as that from the cerebrum and the testicles, should fulfil a large number of clinical indications.—*La Clinica Veterinaria.*

STRYCHNINE POISONING TREATED SUCCESSFULLY WITH ATROPINE AND MORPHINE. The dog, weighing fifteen pounds, had all the symptoms of strychnine intoxication. Rancillia administered one-tenth of a grain of valerianate of atropine, which had no effect. He then injected hypodermically, in two divided doses, 2 centigrammes each of sulphate of atropine and sulphate of morphine—toxic doses—which were counteracted by the strychnine in the organism.—*Journ. de Med. Vet. de Lyon.*

---

### GERMAN ABSTRACTS.

IN the *Deutsche Thierarztliche Wochenschrift* for January 18, 1902, there is an article by Edwin Klebs and Rievel entitled "Is Bovine and Human Tuberculosis Identical."

An experiment is reported wherein a calf was inoculated November 11, 1901, with a culture of tuberculosis growing in bouillon that had been concentrated to one-third of its volume by evaporation in a vacuum at a temperature of from 30° to 40° C. 2.5 c.c. of this material was mixed with an equal volume of finely pulverized wood charcoal and was inoculated beneath the skin of the neck. A similar inoculation was made on the hind leg, and a third inoculation with an equal amount of material was made into the peritoneal cavity. The culture used in this experiment was obtained from a man, and had been grown for several years in Klebs' laboratory in glycerin bouillon. The calf used for the inoculation was twelve weeks old. Its temperature at the time of injection was 39.7° C. The temperature increased to above 40° a short time after the inoculation. The animal was fed boiled milk, hay, and meal. The lymphatic glands about the point of inoculation became enlarged, otherwise the animal did not appear ill. On November 29 it died suddenly. This was eighteen days after inoculation.

On post-mortem examination it was found that there was extensive infection of the lymphatic glands of the neck and shoulder, also of the hind leg. The tissues around the lymphatic glands were congested. There was localized peritonitis and some enlargement of the lymphatic glands within the abdomen. The glands of the stomach and of the mesentery were swollen and of a dark color. The cut surface was moist and contained considerable black material.

The omentum for an area of 30 by 18 cm. showed considerable injection of the bloodvessels, distinct redness, a net-like deposit of grayish-red, flocculent and thread-like tissue that was closely adherent to the omentum. Microscopical examination and the inoculations of guinea-pigs proved that *all* of these lesions were tubercular.

To this experimental case the following cases are added:

A young, healthy man was employed by one of the authors (Klebs) to assist in making some investigations on milk infection. This young man had the habit of drinking milk from the tuberculous cows used in the investigation, and in a few months he died of miliary tuberculosis. The other case, in a family where there were six boys, one died at the age of two years of tuberculosis of the spinal column and the meninges. This child was the only one of the family that developed tuberculosis and also the only one that was fed upon cow's milk.

